# Progress in Skin Diseases

**Published:** 1902-09-27

**Authors:** 


### PROGRESS IN SKIN DISEASES.

**Dermatoses in Special Diseases.**—Enema rashes may be difficult to diagnose from those of septic or infectious diseases, or drug and serum eruptions. Charles Bolton[1] finds that if soft soap is used the chance of such a rash is greatly reduced. After the use of 407 soft soap enemata not a single rash appeared, while after 496 ones of hard soap he noted 17 cases, or 3½ per cent. The rash invariably came out on the day following the injection and lasted from one to three days. It was composed of fine papules, either scattered or in patches, with areas of erythema and sometimes wheals. In some cases it was universal and in others confined to the buttocks, or the trunk, or to the extensor surfaces of the limbs.

**In Bright's Disease.**—Galloway[2] notes two classes of eruptions. The first comprises unimportant sweat rashes, such as erythema and miliaria, caused by baths or medicines, which sometimes lead to dermatitis. Here simple astringent or antiseptic lotions, but not greasy ones, are useful. The second type is of serious import and consists of an erythematous rash with urticarial patches and much desquamation, which may go on to dermatitis exfoliativa. It appears to depend on a vaso-motor paralysis of the capillaries with effusion into the upper layers of the cutis.

**In Diabetes.**—Circinate scaly rashes, often called seborrhœic dermatitis, appear especially in stout persons, and may lead to severe itching and dermatitis. Careful diet has a marked effect in these cases, and weak sulphur and alkaline baths should be given, and the skin after the bath should be dressed with a calamine lotion. Dermatitis of the urinary organs is here very common, and must be treated with antiseptic and astringent lotions. In females, warm baths should be followed by lotions. Those containing 20 to 50 per cent. of glycerine, and $\frac{1}{2}$ to 1 per cent. of carbolic acid are the most useful. Sometimes antipyrin and chloral hydrate given by the mouth aid the recovery.

**Low Tension Lividity,** or a permanently flushed condition of the skin, without definite heart changes, is sometimes seen, and these patients suffer from ulceration after the slightest injuries. A similar state of things occurs with some of the so-called tuberculides. There may be passive localised congestion and infiltration of the subcutaneous tissues in both kinds of disease. Instances are seen in Bazin's disease and acne scrophulosorum. General tonic treatment and possibly ergot should be employed.

**Drug Rashes.**—Stengel[3] notices that purpura is an uncommon result of taking *iodides*, and that the cases are often put down to syphilis. Sometimes the lower limbs are chiefly affected, without any other symptoms, or there may be coryza, hæmorrhages, or a febrile state. The rash may come out in crops, and a continuance of the drug will cause a relapse. He has seen tremor of the limbs, tachycardia, with spongy gums and swelling of the joints and thyroid in these cases. H. C. Wood, jun.,[4] has collected 60 cases of *quinine rashes*, and reports an instance where one appeared after only two doses of half a grain each had been taken. This consisted of regular red spots on both sides of the arms, surrounded by a slight macular rash. There was slight itching and some desquamation afterwards. Workers in quinine also suffer from eruptions. The pathology seems to be either a vaso-motor paralysis or an irritation produced by the excretion of quinine through the skin. The most common forms of a quinine rash are the erythematous and scarlatiniform.

**Alopecia Areata.**—David Walsh[5] records four cases combined with the presence of moniliform hairs. Well marked monilithrix of congenital origin is sometimes found combined with a condition of short scanty hair both on the scalp and the body, so that the patients are more or less bald. The present patients had abundant hair with patches of alopecia, and the moniliform state only occurred in those hairs which were turning white, and did not affect those on the body. He argues that moniliform hair may sometimes be acquired and associated with alopecia, possibly from a weakening of the shaft of the hair by the toxins which cause the baldness. Balmanno Squire[6] reports the cure of a case of universal alopecia which the patient treated daily for 13 months by rubbing in an ointment containing hydrargyri iodidum rubrum, 20 grains to the ounce. This was applied to an eighth part of the scalp each day, and not till after six months did any hair appear. The eyebrows, cheeks, lips, and a large part of the scalp were at last covered with dark hair. He describes the case as it is extremely rare for any improvement to be effected in universal alopecia under any treatment.

**Ringworm.**—G. T. Jackson[7] recommends an application for the scalp in children which has given him excellent results. A drachm of iodine is dissolved in an ounce of goose oil. This is rubbed in twice a day till irritation is produced, and then once a day. If necessary it is stopped for some days. The patch of hair falls off, and the place looks like alopecia, but soon a new growth takes place, and this is a sign of cure. No epilation is necessary. Salicylated oil may be substituted for a time if the reaction is severe. A cure usually results in three weeks. For the few cases which resist this treatment he relies on sulphur ointment containing half a drachm to a drachm of croton oil.

[1] Clin. Jour., Jan. 1. [2] Brit. Med. Jour., May 3. [3] Edin. Med. Jour., April. [4] Ibid. [5] Brit. Med. Jour., April 12. [6] Ibid. [7] Med. Times and Hos. Gaz., June 28.

(*To be continued.*)

